# Draft genome sequence of *Barnettozyma* sp. NCYC 4493 from a UK industrial source

**DOI:** 10.1128/mra.00270-26

**Published:** 2026-09-01

**Authors:** Adrian Turner, Dave J. Baker, Sumeet K. Tiwari, Sam Haynes, Steve A. James, Carmen Nueno Palop

**Affiliations:** 1 National Collection of Yeast Cultures, Quadram Institute Bioscience, Norwich Research Park 455075, Norwich, United Kingdom; 2 Quadram Institute Bioscience, Norwich Research Park 455075, Norwich, United Kingdom; 3 Centre for Microbial Interactions, Quadram Institute Bioscience 7308, Norwich, United Kingdom; University of California Riverside, Riverside, California, USA

**Keywords:** novel, species, *Barnettozyma*, NCYC, 4493, Quadram, institute, national, collection, yeast

## Abstract

*Barnettozyma* species are members of the order *Phaffomycetales*, which include industrially important fungi. A species having ribosomal DNA similarity to *Barnettozyma pratensis*, but distinct from it, was isolated during collaborative identification work for a UK industrial source. Here, we report the draft genome sequence of *Barnettozyma* sp. NCYC 4493.

## ANNOUNCEMENT

During a collaboration with a UK drinks packaging plant, yeast colonies from an agar streak plate produced at the plant and submitted for identification were purified by replating onto Yeast-Mold (YM) agar and Rose Bengal agar supplemented with chloramphenicol (10 mg/mL) and grown at 25°C. Isolates were re-plated until morphologically uniform and stable. DNA was extracted using the MP Bio Soil DNA Extraction Kit, and the D1-D2 region of the large-subunit RNA gene (LSU) was amplified by PCR and sequenced for species identification using primers NL1 (5′-GCATATCAATAAGCGGAGGAAAAG-3′) and NL4 (5′-GGTCCGTGTTTCAAGACGG-3′) (1). A single-colony isolate, coded D6427D, showed no highly similar percentage identity with other LSU rDNA D1-D2 sequences present in the US National Center for Biotechnology Information (NCBI) and European Bioinformatics Institute (EBI) databases, as determined by BLAST searching (2). The highest percentage match was with accession KY106712.1, *Candida qinlingensis* CBS 9768^T^, at 93.1% identity (100% coverage). The isolate was propagated in YM broth for 72 h at 25°C and deposited in the NCYC open collection as NCYC 4493. The D1-D2 sequence was submitted to GenBank as accession number OZ482519.

Genomic DNA was extracted from a 10-mL stationary-phase YM broth culture (grown for 72 h at 25°C) using a FastDNA SPIN Kit for Soil (MP Bio). A short-read library was prepared using a Nextera DNA Flex Library Prep Kit (Illumina) with 20-fold diluted library reagents. Sequencing was performed on a NextSeq 2000 sequencer, producing 1,809,210 paired-end 150-bp reads (~38× coverage).

A Nanopore library was prepared from the same gDNA prep, without shearing or size selection, using the 1D Ligation Kit (catalog number SQK-LSK109; Oxford Nanopore Technologies, ONT), and loaded onto a FLO-PRO114M flow cell (ONT) on a PromethION 2 Solo instrument (ONT). This produced a total of 45,133 reads with an average read length of 4,535 bp (14.4× coverage). Default parameters were used for all software unless otherwise specified. Base calling was performed using MinKNOW (ONT; v24.11.8) in super-accuracy mode (model dna_r10.4.1_e8.2_400bps_sup@v4.3.0). Raw short- and long-read trimming and filtering were performed using fastp v1.0.1 and fastplong v0.4.1 (3).

*De novo* assembly with Nanopore reads was performed using Flye 2.9.6 (4), using long reads in the “ONT high-quality” mode. The assembly was polished first with long reads using medaka v2.0.0 (ONT) and then with the Illumina data using NextPolish v1.4.1 (5). Assembly statistics were generated using QUAST v.5.0.2 (6). The assembly consists of 29 contigs, and the total length is 11,166,610 bp, with an N50 value of 1,104,533 bp and a G + C content of 39.35%. Genome completeness was estimated as 95.2% using BUSCO (QIB Galaxy v.5.5.0) (7, 8) with the saccharomycete_odb10 data set. Augustus (QIB Galaxy v.3.2.3) (8, 9) predicted 5,389 protein-coding genes using the *Saccharomyces cerevisiae* training data set.

Barrnap (QIB Galaxy v.1.2.1) (8, 10) was used to extract the 18S rDNA region from the assembly. Three copies were identified, located on contigs 14, 15, and 30. The closest species match with other 18S rDNA sequences present in the NCBI and EBI databases, as determined by BLAST searching, was *Barnettozyma pratensis* NRRL Y-12696 (NG_063293.1), with 97.85% identity (93% coverage).

## Data Availability

This whole-genome shotgun project has been deposited in ENA (BioProject number PRJEB114506, assembly accession number GCA_987087495). The raw reads are available in the EBI database under accession numbers ERR17587779 (ONT) and ERR17373641 (Illumina). The Augustus gene prediction set is publicly deposited in Zenodo at https://doi.org/10.5281/zenodo.22030059.
